# Effect of Biofilm Formation by *Oenococcus oeni* on Malolactic Fermentation and the Release of Aromatic Compounds in Wine

**DOI:** 10.3389/fmicb.2016.00613

**Published:** 2016-04-27

**Authors:** Alexandre Bastard, Christian Coelho, Romain Briandet, Alexis Canette, Régis Gougeon, Hervé Alexandre, Jean Guzzo, Stéphanie Weidmann

**Affiliations:** ^1^UMR A PAM Université Bourgogne Franche-Comté – AgroSup Dijon – Equipe Vin, Aliment, MicrobiologieDijon, France; ^2^UMR A PAM Université Bourgogne Franche-Comté – AgroSup Dijon – Equipe Procédés Alimentaires et Physico-ChimieDijon, France; ^3^Micalis Institute, INRA, AgroParisTech, Université Paris-SaclayJouy-en-Josas, France

**Keywords:** malolactic fermentation, *Oenococcus oeni*, biofilm, wine, oak

## Abstract

The winemaking process involves the alcoholic fermentation of must, often followed by malolactic fermentation (MLF). The latter, mainly carried out by the lactic acid bacterium *Oenococcus oeni*, is used to improve wine quality when acidity reduction is required. Moreover, it prevents microbial spoilage and improves the wine’s organoleptic profile. Prior observations showed that *O. oeni* is able to resist several months in harsh wine conditions when adhered on oak barrels. Since biofilm is a prevailing microbial lifestyle in natural environments, the capacity of *O. oeni* to form biofilms was investigated on winemaking material such as stainless steel and oak chips. Scanning Electron Microscopy and Confocal Laser Scanning Microscopy showed that *O. oeni* was able to adhere to these surfaces and form spatially organized microcolonies embedded in extracellular substances. To assess the competitive advantage of this mode of life in wine, the properties of biofilm and planktonic cells were compared after inoculation in a fermented must (pH 3.5 or 3.2 and 12% ethanol) The results indicated that the biofilm culture of *O. oeni* conferred (i) increased tolerance to wine stress, and (ii) functional performance with effective malolactic activities. Relative gene expression focusing on stress genes and genes involved in EPS synthesis was investigated in a mature biofilm and emphasized the role of the matrix in increased biofilm resistance. As oak is commonly used in wine aging, we focused on the *O. oeni* biofilm on this material and its contribution to the development of wine color and the release of aromatic compounds. Analytical chromatography was used to target the main oak aging compounds such as vanillin, gaiacol, eugenol, whisky-lactones, and furfural. The results reveal that *O. oeni* biofilm developed on oak can modulate the wood-wine transfer of volatile aromatic compounds during MLF and aging by decreasing furfural, gaiacol, and eugenol in particular. This work showed that *O. oeni* forms biofilms consisting of stress-tolerant cells capable of efficient MLF under winemaking conditions. Therefore surface-associated behaviors should be considered in the development of improved strategies for the control of MLF in wine.

## Introduction

The winemaking process involves the alcoholic fermentation (AF) of must performed by yeast, often followed by malolactic fermentation (MLF) performed by lactic acid bacteria (LAB). MLF is involved in the quality of red, white, and sparkling wines, for which it is necessary to reduce acidity (cool-climate regions). MLF also prevents microbial spoilage through nutrient consumption (sugars, malic acid) and the release of aromatic compounds that improve the organoleptic profile of wine (Bauer and Dicks, 2004). MLF is not in itself a fermentation process but rather the decarboxylation of L-malate (di-acid) into L-lactate (mono-acid) and CO_2_ by the malolactic enzyme (MLE). This reaction allows cells to regulate their internal pH and gain energy through the proton gradient across cell membranes (Versari et al., 1999).

Several LAB genera including *Lactobacillus*, *Leuconostoc*, *Pediococcus*, and *Oenococcus* are able to decarboxylate L-malate. Of the latter *Oenococcus oeni* appears best able to maintain its metabolism in an environment with low pH (ca. 3.5) and the presence of SO_2_ (Vuuren and Dicks, 1993; Lonvaud-Funel, 1999). This bacterium can convert malic acid in a one-step reaction (Lonvaud-Funel and Strasser de Saad, 1982; Salou et al., 1994; Kourkoutas et al., 2004). Furthermore, MLF driven by *O. oeni* leads to improving the organoleptic properties and microbiological stability of wine, through residual sugar consumption, the bacterial fermentation of co-products and lactic acid production (Lonvaud-Funel, 1995; Nedovic et al., 2000). However, despite the efficiency of *O. oeni*, spontaneous MLF is difficult to predict. Several physicochemical parameters of wine such as ethanol, low pH, and the presence of sulfite can delay MLF. Winemakers increasingly need to control their production, therefore the use of commercial starter cultures to induce MLF has become common practice. However, because of the rapid loss of cell viability after inoculation, the result is not always successful (Bauer and Dicks, 2004). Other solutions have been sought. For instance, the gene encoding the MLE of *O. oeni* is expressed in genetically modified microorganisms such as *Lactobacillus plantarum* and *Saccharomyces cerevisiae*, but few countries allow GMOs for food processing purposes (Schümann et al., 2012). Likewise, the yeast *Schizosaccharomyces pombe* was studied since it can convert malic acid through malo-ethanolic fermentation. Nevertheless, it increases ethanol levels and provides no beneficial aspects for MLF (Ansanay et al., 1996; Versari et al., 1999). It has been shown that MLF does not necessarily require cell growth: non-proliferating cells of *O. oeni* at 10^6^ to 10^7^ CFU/ml can decarboxylate malic acid (Lafon-Lafourcade, 1970). These results suggest that, as described in previous works for other alcoholic fermented beverages, surface-associated cells could be used to perform MLF (Nedovic et al., 2000; Kourkoutas et al., 2004; Brányik et al., 2005; Genisheva Z. et al., 2014; Genisheva Z. A. et al., 2014; Nedović et al., 2015).

The capacity of *O. oeni* to compete in a harsh environment such as wine is due to elaborate survival strategies of which we can mention the adjustment of membrane stability by changing the ratio of saturated-unsaturated fatty acids (Grandvalet et al., 2008; Maitre et al., 2014), and the synthesis of stress proteins (Jobin et al., 1997; Guzzo et al., 2000; Beltramo et al., 2006; Maitre et al., 2012). In addition, *O. oeni* can adapt to ethanol stress, especially *via* the synthesis of the small heat shock protein Lo18 (Jobin et al., 1997; Coucheney et al., 2005; Maitre et al., 2012, 2014). Biofilm formation is another way of resisting environmental stresses. This process has been widely described for bacteria, since it represents the dominant mode of microbial existence (Costerton et al., 1995). A biofilm is a community of microorganisms bound together in close proximity within their own protecting exo-polymeric matrix, permitting metabolic cross-feeding, cell–cell interactions and chemical and physical resistance (Davey and O’toole, 2000; Hojo et al., 2009). Due to this specific organization, the biofilm is considered as a whole (Katharios-Lanwermeyer et al., 2014). The biofilm formation of the lactic acid bacterium *Lb. plantarum* biofilm enhances stress resistance to acetic acid (up to 11% v/v) and ethanol (up to 40% v/v). Indeed, the analysis of cell surfaces by scanning electron microscopy (SEM) revealed that that these treatments severely damage planktonic cells whereas biofilm cells were only slightly damaged (Kubota et al., 2008). Many examples of transformation processes using biofilm on the laboratory scale have been documented, such as wastewater treatment and ethanol production, but so far the only industrial application of biofilms for food production purposes known to date is the production of acetic acid by acetic acid bacteria biofilm (Maksimova, 2014).

Up to now, very little attention has been given to *O. oeni* biofilm formation, and only its bacteriocin resistance properties have been reported (Nel et al., 2002). However, a connection has been reported between *O. oeni* EPS production and its increased survival in wine (Dimopoulou et al., 2015).

In a previous experiment, the sampling of oak barrels suggested that microorganisms and particularly LAB were able to withstand wine stress (low pH, ethanol, few nutrients) on this surface. Thus in this context, our study investigated the surface-associated behaviors of *O. oeni* cells and their role in resistance to stresses incurred in wine. We examined the spatial organization of *O. oeni* cells on different contact surfaces, the survival of surface-associated cells, and their ability to perform MLF in wine. Finally, we explored the impact of oak surface-associated *O. oeni* cells on the color and aromatic profile of wine in view of the importance of this material in winemaking and aging.

## Materials and Methods

### Bacteria Strains and Growth Media

This study was conducted using two strains: ATCC-BAA 1163, one of the first strains of *O. oeni* to be sequenced (isolated from red wine, France, Aquitaine) and currently used as a reference (Guzzo et al., 2000; Beltramo et al., 2004; Desroche et al., 2005; Maitre et al., 2012), and Sabo11, an enological strain (isolated from red wine, South Africa) presenting enhanced technological properties and currently used at the *Domaine viticole de l’Université de Bourgogne, Marsannay, France* to perform MLF. Bacteria were grown in MRS modified (MRSm) medium containing: MRS Broth (Laboratorios Conda Spain) 50 g/l; fructose 10 g/l; L-malic acid 4 g/l. The pH was adjusted to 4.8 (NaOH concentrated solution). For solid MRSm medium, 25 g/l agar was added.

Wine medium was obtained by the fermentation of a commercial white grape juice by commercial yeast (*Saccharomyces cerevisiae* Fermol PB 2023, Spindal AEB Group). The outcome was standardized at 12% ethanol, pH3.2 or 3.5, fermentable sugars 2 g/l and L-malic acid 4 g/l.

Aligoté white wine from the 2014 vintage elaborated at the *Domaine viticole de l’Université de Bourgogne, Marsannay, France*, was used for aroma analysis. This wine finished its alcoholic fermentation with the following enological parameters: 12% ethanol, pH 3.5, and L-malic acid 3.2 g/l.

All the media were sterilized by filtration (0.2 μm cut-off). Cultures were incubated at 28°C with 10% CO_2_ in a CO_2_ incubator. All the assays were performed in triplicate.

### Biofilm Formation Conditions

#### On Stainless Steel Chips

Each 25 mm × 25 mm stainless-steel chip (Goodfellow) was immersed in 20 ml inoculated MRSm (2 × 10^7^ CFU/ml). After incubation for 3, 7, and 14 days (with a medium turnover every 3.5 days), the plate was rinsed twice with NaCl 150 mM, then placed in 10 ml saline solution with 700 mg of 0.1 mm diameter glass beads. The system was vortexed at maximal power for 2 min to free surface-associated cells. Populations of cells removed from the surface by this procedure were estimated by culturing appropriate dilutions (prepared in NaCl 150 mM) on solid MRSm at 28°C under 10% CO_2_. It was previously verified that the bead treatments dislodged surface-associated cells and did not cause cell death, by measurement of viable planktonic cell populations before and after these treatments. The 2-week-old biofilm was detached from the steel plate into the wine to assess biofilm cell viability after 1, 4, and 24 h.

#### On Oak Chips

The oak wood used in this study was characterized by a previous work (Duval et al., 2013). The 25 mm × 25 mm oak chips were immersed in 20 ml of inoculated MRSm (2 × 10^7^ CFU/ml). The medium was changed every 3 days until the end of incubation (1, 2, or 4 weeks). Surface-associated cell populations were estimated as follows. The chips were rinsed twice with sterile saline solution, placed in 10 ml saline and scrubbed with a toothbrush (2 min per side). Viable cell populations in this solution were determined on solid MRSm medium as described above.

To analyze biofilm survival in wine, the chips were rinsed twice with saline solution, transferred to wine and incubated for 1, 4, 7, 14, or 21 days. Their populations were estimated as described above. All the assays were performed in triplicate.

#### On a Polystyrene Microplate

Two hundred and fifty micro liter of a mid-exponential phase culture (10^9^ CFU/ml) was added to the wells of a polystyrene 96-well microtiter plate (Greiner Bio-one, France) with a μclear^®^ base (Polystyrene, thickness of 190 μm ± 10%) which allowed high resolution confocal imaging. After 1 h of adhesion at 30°C, the wells were refilled with 250 μl MRSm. This preparation was then subjected to Confocal Laser Scanning Microscopy.

### Confocal Laser Scanning Microscopy

Surface-associated microorganisms were fluorescently tagged by adding FM4-64 fluorescent membrane marker (Life Technologies, USA) in fresh medium according to the manufacturer’s instructions. The plate was incubated for 40 h at 30°C and mounted on the motorized stage of an inverted confocal microscope (Leica SP8 AOBS, LEICA Microsystems, Germany) at the INRA-MIMA2 imaging platform^1^. Observations were performed using a 63X/1.2 N.A. water immersion objective lens (300 μm working distance). Surface-associated microbial agglomerates were scanned using an argon gas laser with a 514 nm line (output power at 30%, AOTF at 10%) and the fluorescence emitted was recorded from 534 to 800 nm using a PMT detector with a gain of 750 V. Single 2D sections of surface-associated agglomerates and 3D acquisitions were acquired at a scan speed of 600 Hz an image definition of 512 × 512 and a z-step of 1 μm between each xy image for a z-stack. Time-lapse automated acquisitions were performed with the LAS X High Content Screening A Matrix Screener module. Three-dimensional projections of agglomerate structure were then reconstructed using the blend mode of the Easy 3D function of the IMARIS 7.7.2 software (Bitplane, Switzerland). Microbial agglomerate biovolumes (μm^3^) were extracted from confocal image series using a homemade ICY routine as described previously (Sanchez-Vizuete et al., 2015).

### Scanning Electron Microscopy

Cells were fixed on stainless steel by a solution of 2.5% glutaraldehyde in 0.1 M phosphate buffer pH 7.2 for 1 h at 4°C. The samples were then washed three times with phosphate buffer for 20 min at room temperature. Dehydration was performed by successive immersions in solutions of increasing ethanol content (70, 90, 100%), then three times for 10 min each in successive baths of ethanol-acetone solution (70:30, 50:50, 30:70, 100) and air-dried. Afterward, the samples were coated with a thin carbon layer using a CRESSINGTON 308R and observed with a JEOL JSM 7600F scanning electron microscope (JEOL, Ltd.). SEM was performed at 5 kV and the samples were observed at a working distance of 14.9 mm.

### Malolactic Conversion Monitoring

Malolactic fermentation monitoring was performed according to the manufacturer’s instructions using the “L-Malic acid Cat No. 020” kit from Biosentec.

### Gene Expression Analysis

#### RNA Extraction and cDNA Preparation

Planktonic cells were sampled in the mid-exponential phase and the surface-associated cells after 2-weeks growth on steel. Cells were centrifuged (8,000 *g*, 10 min) before being resuspended in 1 ml of Tri-reagent (Sigma) and disrupted with glass beads (100 μm) in a Precellys homogenizer (Bertin) for 6 series of 30 s at 6500 rpm. Nucleic acids were extracted in 0.2 volume of chloroform and purified by precipitation in 1 volume of isopropanol. RNA pellets were dried and resuspended in 30 μl of RNase-free water. Nucleic acid concentrations were calculated by measuring absorbance at 260 nm using an Infinite 200 PRO spectrophotometer (Tecan). Before reverse transcription (RT), 2 μg of total RNA were treated with 2 U of DNase (Invitrogen), as described by the manufacturer. The absence of chromosomal DNA contamination was checked by real-time PCR. cDNAs were then synthesized by using an iScript cDNA synthesis kit (Bio-Rad) as recommended.

#### Real-time PCR Experiment

Real-time PCR as described by Desroche et al. (2005) was used to quantify mRNA levels. Gene specific primers (**Table 1**) were designed to amplify the cDNAs of the transcripts of *ldhD, gyrA, hsp18, clpL1, cfa, groEL, levO, wobB, wobO, dsrO, mleA* with the Bio-Rad SYBR green kit in a Bio-Rad I-Cycler. This method was used to analyze their mRNA levels during planktonic growth at mid-exponential phase (10^9^ CFU/ml) and 2-weeks of biofilm development on stainless-steel chips (2 × 10^6^ CFU/cm^2^) with or without wine stress (pH 3.5; 12% ethanol). The results were analyzed by using a comparative critical threshold method (ΔΔCT) in which the amount of targeted mRNA was first normalized using both the specific mRNA standard and then compared to a calibrator condition (Desroche et al., 2005). *ldh* and *gyrA* genes encoding for a glyceraldheyde-3-phosphate dehydrogenase and a gyrase, respectively, were selected as internal standards since their transcript levels were stable under the conditions tested. mRNA quantification was performed in triplicate from the total RNA extracted from three independent cultures.

**Table 1:** Primers used for gene expression analysis.

Target gene	Function of gene	Forward primer (5′→3′)	Reverse primer (5’→3’)	Amplicon length (bp)	Reference
*cfa*	Cyclopropane fatty acid synthase	GGTATTACATTGAGCGAGGAG	CGTCTTTGAGATCACGATAATCC	113	Beltramo et al., 2006
*clpL1*	Clp ATPase protein	ATTATAATGACGATCCCTTCGT	GGATCCCTGAACCGTTATTTGCTTGTTG	163	Desroche et al., 2005
*dsrO*	Glycoside-hydrolase	GGTCGCTGCTGCTTAATTTC	CCGTGGTGTTTTGACATCAG	137	This study
*groEL*	Heat shock chaperone	TCCCACGAAGTTGAGGATTC	CGATACCTTTGGACTCTTCA	145	This study
*gyrA*	Gyrase α subunit	CAAGGACTCATAGATTGCCGAA	CGCCCGACAAACCGCATAAA	95	Desroche et al., 2005
*hsp18*	sHsp Lo18	CGGTATCAGGAGTTTTGAGTTC	CGTAGTAACTGCGGGAGTAATTC	102	Beltramo et al., 2006
*ldhD*	D-lactate dehydrogenase	GCCGCAGTAAAGAACTTGATG	TGCCGACAACACCAACTGTTT	102	Desroche et al., 2005
*mleA*	Malolactic enzyme (MLE)	CCGACAATTGCTGATACAATTGAA	GGCATCAGAAACGACCAGCAG	156	Beltramo et al., 2006
*levO*	Fructansucrase	AATCAAGATACCGCCAGTGC	CCGAACCTGACCATTGTTCT	109	This study
*wobB*	Rhamnosyl-transferase	TGGTACAAATCGACCGACAA	AAAGTCCGTGATTGGTTTGC	75	This study
*wobO*	Glycosyltransferase	TGTCGAATGGAACATGAACG	TGATCGTCTCGATGATTGGA	62	This study

### Measurement of Oak Aroma Compounds Released in Wine by HS-SPME-GC-MS

HS-SPME-GC-MS was carried out using the method of Duval et al. (2013). Five ml of 1-month old wine was placed in a 20 ml sealed headspace vial (Supelco, Bellefonte, PA, USA). Headspace vials were then placed in the agitator/incubator of an automatic headspace sampler (GERSTEL MPS 2, Gerstel Inc., Mülheim an der Ruhr, Germany) and incubated at 70°C for 10 min (incubation time) in order to promote volatile compounds in the headspace. Extractions were performed by immersing a DVB–CAR–PDMS fiber in the headspace for 60 min (extraction time). After each extraction, the extracted compounds were desorbed at 260°C for 7 min in the injection port of an HP 6890GC equipped with an MSD 5973 mass detector (Agilent Technologies, Palo Alto, CA, USA). Calibration solutions were processed in the same way using 5 ml of the wine matrix mixed with target compounds. Volatile compounds (eugenol, guaiacol, furfural, vanillin, cis-, and trans-whisky lactone) were purchased from Sigma–Aldrich and used as received. We used 3,4-dimethylphenol as the internal standard at 10 mg/l in each sample. Using highly aroma-concentrated calibration samples either alone or in mixture, we checked that there were no competition effects for the fiber between aromas. Chromatographic analyses were performed in biological triplicate and technical duplicate.

#### Chromatographic Conditions

The oven program started at an initial temperature of 40°C for 3 min. The temperature was then increased at a rate of 7°C min^–1^ up to 230°C. A 0.8 mm I.D. liner was used and maintained at 270°C, in splitless injection mode. The carrier gas was helium at 1.0 ml.min^–1^ (99.996%). Ionization was performed by electronic impact (EI), with the electron multiplier set at 1600 eV. The temperatures used were 200°C for the trap, 60°C for the manifold, and 280°C for the transfer line. The compounds were quantified in selected ion storage (SIS) mode, by selecting the appropriate ion masses for each compound: furfural (95 + 96), guaiacol (109 + 124), whisky lactone (99), eugenol (164), 3,4-dimethylphenol (107 + 122), vanillin (151 + 152).

#### Color Measurements

Color absorbance measurements and data acquisition and analysis were performed with a Konica Minolta CM-5 spectrophotometer using optical glass precision cells with a 50 mm path length (Hellma Analytics) and scanned over the range 740–360 nm (visible range). Black and white calibrations were performed using a standard black plate and an empty glass cell, respectively. Color was recorded using the CIE-L^∗^ a^∗^ b^∗^ uniform color space (CIE-Lab), using three dimensions (L^∗^, a^∗^, b^∗^) of the Hunter color scale, where L^∗^ ranges from 0 for black to +100 for white, a^∗^ ranges from –50 for green to +50 for red, and b^∗^ ranges from –50 for blue to +50 for yellow.

### Statistical Analysis

Each experiment was carried out in triplicate. Error bars represent standard deviations. Student *t*-test and one-way analysis of variance (ANOVA) followed by a Tukey’s HSD (honest significant difference) post hoc test were used to analyze significant differences between groups using XLSTAT Version 2014, Addinsoft; *P* = 0.05. Principal Component Analysis of data was carried out with the same software.

## Results

### *Oenococcus oeni* Can Colonize Different Surfaces

**FIGURE 1:**
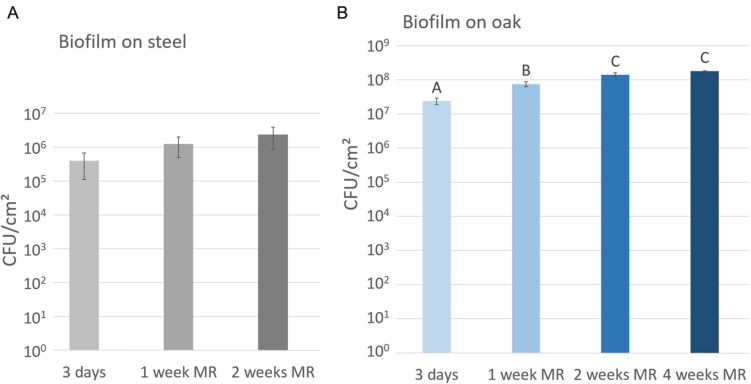
**Biofilm growth of *Oenococcus oeni* ATCC BAA-1163 in MRSm renewed twice a week (MR) on **(A)** stainless steel or **(B)** oak**. Biomass is expressed in CFU/cm^2^. Error bars represent the standard deviation of three biological replicates; a different letter means significant difference *P* < 0.05.

Stainless steel tanks and oak barrels are used in winemaking, therefore the development of *O. oeni* was characterized on both surfaces. An *O. oeni* ATCC BAA-1163 population grown on a stainless steel chip was numbered after 3 days, 1 and 2 weeks, respectively (**Figure 1A**). On stainless steel the surface-associated cells reached 4 × 10^5^ CFU/cm^2^ in 3 days. At 1 week, they reached a population of almost 10^6^ CFU/cm^2^ and then exceeded it after 2 weeks (2 × 10^6^ CFU/cm^2^) (**Figure 1A**).

On oak, surface-associated cells were around 60-fold more numerous than on steel with a population reaching 2 × 10^7^ CFU/cm^2^ and 10^8^ CFU/cm^2^ at 3 days and 2 weeks, respectively (**Figure 1B**). The growth of these cells slowed down from the 2nd week and the population remained constant.

**FIGURE 2:**
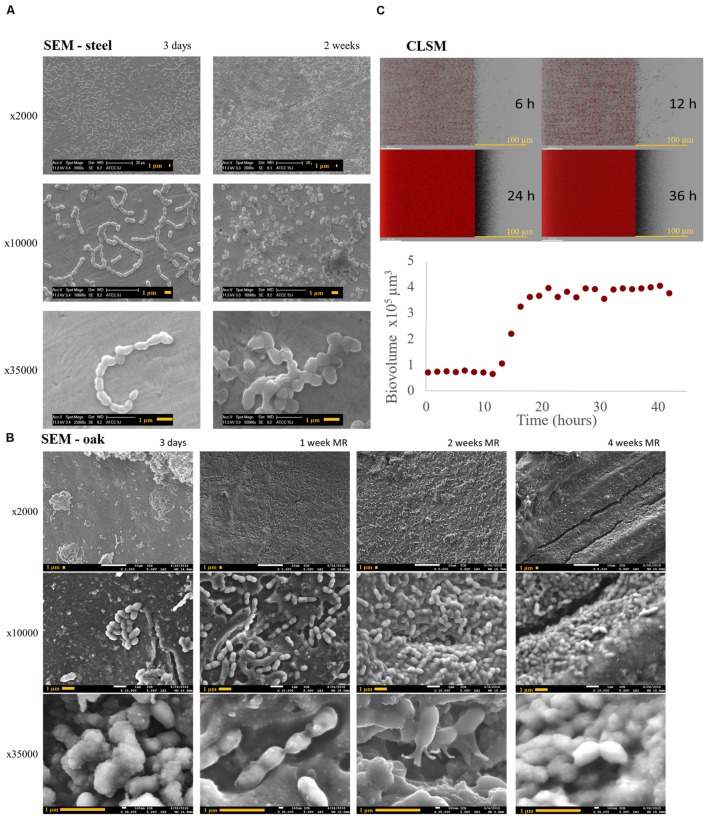
***Oenococcus oeni* ATCC BAA-1163 biofilm microscopy observations. (A,B)** Scanning Electron Microscopy (SEM) at x2000 x10,000 x35,000 of biofilm growth showing stages of formation **(A)** on steel at 3 days and 2-weeks’ growth, and **(B)** oak at 3 days, 1, 2, and 4 weeks. **(C)** Confocal Laser Scanning Microscopy z-projections for the time lapse of biofilm development at 6, 12, 24, and 36 h on polystyrene microplates. Below, the evolution of the biofilm biovolume.

The difference between the populations studied on steel and oak was confirmed by SEM observation (**Figures 2A,B**). Although it did not cover the entire surface, the tridimensional organization of cells on oak appeared thicker, wider and more mature. The early stages of this tridimensional development were observed at each time on steel (3 days to 2 weeks), showing cell adhesion and microcolonies. The cells adhered, flattened, and produced extracellular material that bonded them to the surface, after which they finally organized themselves in microcolonies (**Figure 2A**). These characteristics observed for the surface-associated cells allowed us to consider that *O. oeni* is able to form a biofilm On oak, there was an observable transition between the 1-week stage and the 2-week growth stage. Indeed, at this point, most of the cells appeared to belong to a larger structure and merged in a matrix (**Figure 2B**).

This matrix was observable as was a polymer that attached the cells to the surface (**Figure 2A**: Steel, 2 weeks, x35 000, and **Figure 2B**: oak, 2 weeks, x35 000), bonded them together (**Figure 2B**: oak, 1 week, x35 000), and coated the surface of the biofilm, so that the cells were indistinguishable (**Figure 2B**: oak, 4 weeks, x35 000). According to these observations, the biofilm appeared mature from 2 weeks on oak.

To gain more insight into *O. oeni* biofilm formation dynamics, we used a Real-Time Confocal Laser Scanning Microscope (RT-CLSM) associated with a fluorescent membrane probe compatible with live *in situ* dynamics to monitor cell growth in 4D over 2 days (**Figure 2A**). Technically, this observation was not possible on wood chips (autofluorescence, non-transparency, interaction with the fluorophore), so measurements were performed in polystyrene microplates. Surface-associated *O. oeni* showed a rapid increase in biovolume, reaching up to 4 × 10^5^ μm^3^ after 18 h incubation (**Figure 2C**).

### *Oenococcus oeni* Biofilm, a Mode of Life Allowing Stress Resistance

**FIGURE 3:**
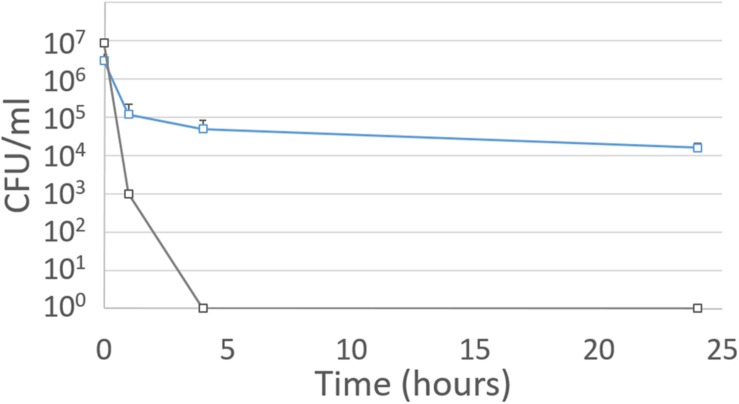
**Evolution of *O. oeni* ATCC BAA-1163 population in wine**. Gray curve: planktonic cells; blue curve: biofilm cells (2-week growth, medium renewed) detached from the steel. The wine used has a pH of 3.2; 12% ethanol. Error bars represent the standard deviation of three biological replicates.

The survival of planktonic and biofilm cells detached for 2 weeks in wine was compared. Both samples were inoculated in wine medium at pH 3.2 with 12% ethanol, which represents severe stress conditions for *O. oeni*. Their survival was monitored for 24 h (**Figure 3**). Planktonic cells inoculated at 10^7^ CFU/ml in this medium underwent total mortality within 4 h, while cells detached from the biofilm (inoculated at 3 × 10^6^ CFU/ml) had a loss of 1 log after 4 h incubation. However, viability remained constant over 24 h (**Figure 3**), suggesting that biofilm cells keep their properties even when detached. This made it possible to describe a real biofilm phenotype for the cells in the microcolonies and the cells detached from the biofilm.

**FIGURE 4:**
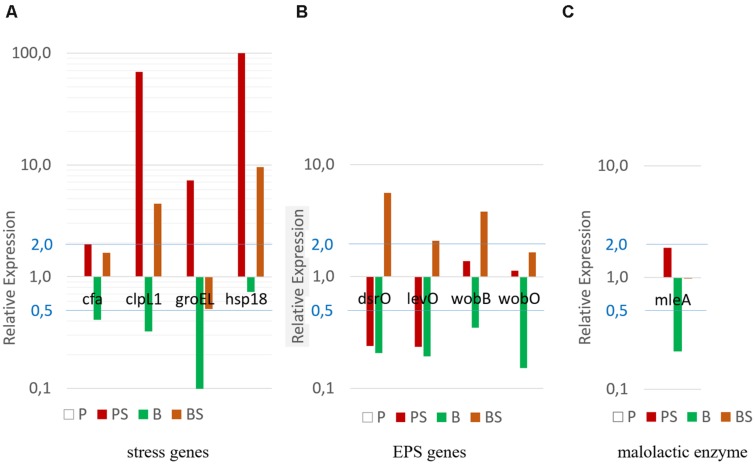
**Comparison of relative expression levels of (A) five stress genes, (B) four genes involved in EPS production, and (C) malolactic enzyme (MLE) gene, of *O. oeni* ATCC BAA-1163 in the planktonic exponential phase and biofilm with 2-weeks’ growth**. Planktonic cell gene expression without stress (P) was used as a calibrator and set at 1; green bars represent biofilm cells without stress **(B)**. Red bars represent planktonic stressed cells (PS), and orange bars stressed biofilm (BS), knowing that each group was exposed to a 30-min wine stress (pH 3.5 ethanol 12%). Gene expression was quantified using RT-qPCR and the comparative critical threshold (ΔΔC_T_) method. The *ldhD* gene was used as the internal control. One representative repetition of the triplicate is shown.

The biofilm phenotype increased cell stress resistance, even after detachment from the surface. In order to investigate biofilm tolerance mechanisms, we studied the relative expression of a set of genes encoding for stress proteins (*hsp18, clpL1, cfa, groEL*) (**Figure 4A**) and a set of genes involved in exopolysaccharide production (*levO, wobB, wobO, dsrO*) (**Figure 4B**), during the biofilm development (2-week old biofilm) and the planktonic growth (exponential phase) with or without stress (30 min in wine at pH 3.5 and ethanol 12%). As expected, genes related to stress response were overexpressed in stressed planktonic (PS) cells compared to non-stressed planktonic cells (P) (**Figure 4A**). The *cfa* transcript level was slightly higher and the *groEL* transcript levels were sixfold higher. The highest increases were for *clpL1* and *hsp18* transcript levels, at approximately 70-fold and 150-fold. Regarding biofilm cells (B), all the genes studied in the non-stressed biofilm showed lower expression compared to the non-stressed planktonic cells (P). However, stress genes were over-expressed (except for *groEL*) when biofilm cells were exposed to stress conditions (BS) (**Figure 4A**).

The relative expression levels of four genes involved in EPS production in planktonic and biofilm cells, with or without stress, are described in **Figure 4B**. In stressed planktonic cells (PS), *dsrO* and *levO* exhibited a fourfold decrease in transcription levels compared to the planktonic reference (P). Expressions of the genes studied and involved in the production of EPS were lower in the non-stressed biofilm cells (B) than in the planktonic reference (P) (2.9-fold to 6.7-fold) (**Figure 4B**). In contrast, when biofilm cells were stressed (BS), the expression of these genes increased significantly (10 times the B levels).

### Impact of Biofilm and Planktonic Cells of *O. oeni* on the Malolactic Fermentation of Wine

Since the biofilm phenotype provides improved stress resistance, biofilm technological performance was investigated in comparison with planktonic cells. To establish whether the biofilm of *O. oeni* keeps its enological properties, the consumption of malic acid was monitored simultaneously with the quantitative analysis of transcript levels of the gene encoding for the malolactic enzyme (*mleA*). As shown in **Figure 4C**, *mleA* is less expressed in biofilm cells (B) compared to exponential-planktonic cells (P). However, when biofilm cells were immersed in wine (BS), their *mleA* transcription levels were similar to planktonic cells (P). Indeed, at the time of sampling malic acid was no longer present in the biofilm culture medium contrary to the wine medium, suggesting that *mleA* transcript level is reletad to the acid malic concentration in the medium.

**FIGURE 5:**
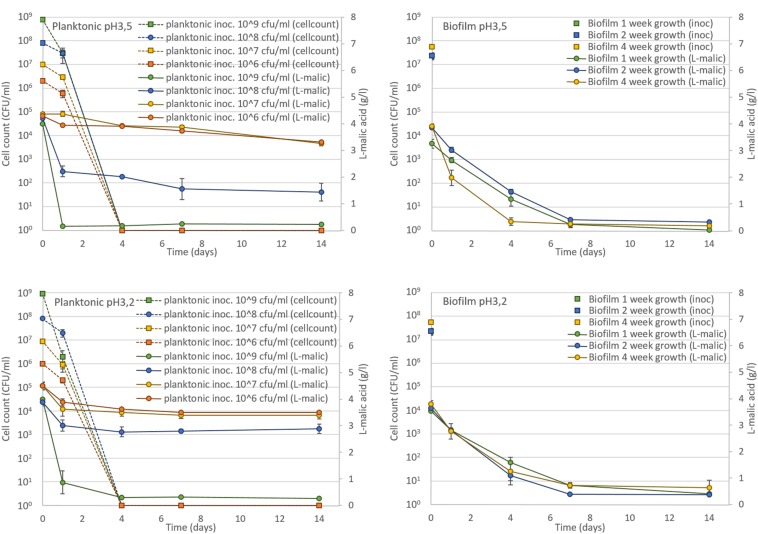
**Comparison of L-malic acid conversion by *O. oeni* ATCC BAA-1163 planktonic (**left** side) and biofilm cells (right side) in wine medium (12% ethanol) at pH3.5 (up), and pH3.2 (bottom)**. Planktonic cells were inoculated from 10^6^ to 10^9^ CFU/ml then their mortality was monitored (dashed lines). Biofilms of 1, 2, 4-weeks’ growth on oak chip were inoculated at the equivalent of 10^8^ CFU/ml. Error bars represent the standard deviation of three biological replicates.

Microvinifications were carried out using a must fermented by *S. cerevisae*, adjusted to pH 3.2 or pH 3.5, 4 g/l L-malic acid and 12% ethanol, inoculated with *O. oeni* ATCC BAA-1163 biofilm on oak at 5 × 10^7^ CFU/ml or planktonic cells as reference (10^6^ to 10^9^ CFU/ml). After 4 days incubation in this wine (**Figure 5**), the planktonic cells underwent total mortality regardless of the initial concentration inoculated, suggesting that without pre-adaptation they are unable to survive in wine and consequently unable to perform MLF. Despite this mortality, a very large cell population (10^9^ CFU/ml) could convert malic acid before dying. In contrast, biofilm cells kept their ability to perform complete MLF, probably due to their enhanced survival in wine (**Figure 5**).

**FIGURE 6:**
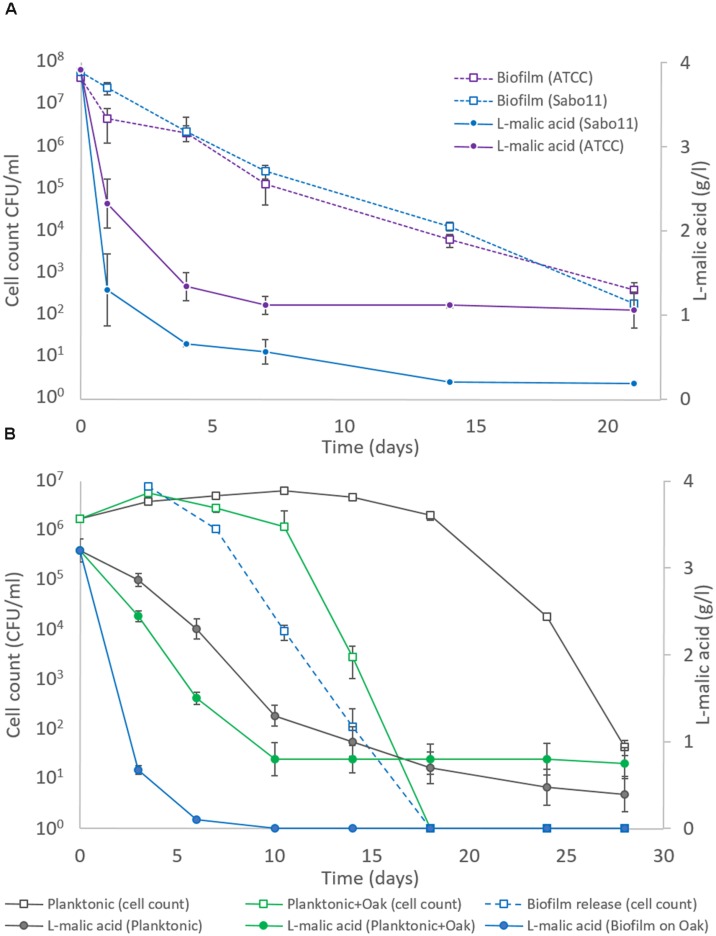
**(A)** Monitoring of the MLF in *aligoté* wine (pH3.5 ethanol 12%) by two *O. oeni* strains grown in biofilm on oak for 2 weeks. Biofilm inoculum and survival are shown by dashed lines (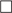 ATCC BAA-1163; 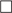
Sabo11). L-malic concentration is shown in straight lines (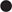 ATCC BAA-1163; 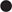
Sabo11). **(B)** Monitoring of MLF (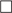) and cell viability (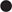) in aligoté wine (pH3.5 ethanol 12%) by a adapted planktonic inoculum of *O. oeni* Sabo11 (gray lines), supplemented with oak chip (green lines), and biofilm on oak chip (blue lines). The blue dashed line represents the viable-cultivable cells released by the biofilm in the wine. Error bars represent the standard deviation of three biological replicates.

Following this strategy, we made a comparison between an *O. oeni* lab strain ATCC BAA-1163 and Sabo11, a malolactic strain of technological interest (**Figure 6A**). Indeed, Sabo11 completed 100% MLF whereas ATCC BAA-1163 converted 75% of the L-malic acid. This difference was not due to the cell quantity, because both populations exhibited the same viability through time, which decreased from 5 × 10^7^ CFU/ml (beginning) to 10^3^ CFU/ml (20 days after). Consequently, Sabo11 was more suitable for performing MLF than the lab strain, ATCC BAA-1163. Therefore this strain was used to perform a winemaking-like experiment involving interaction between bacteria, oak and wine. To this end, a planktonic culture of Sabo11 was adapted to wine stress with the *pied-de-cuve* method (Li et al., 2012). As shown in **Table 2**, we compared five samples in which the presence of oak and the bacteria mode of life vary, in order to test an alternative to traditional wine inoculation through the *pied-de-cuve*. Therefore we used biofilms which were not adapted to wine conditions, unlike the planktonic culture. MLF monitoring in wine is shown in **Figure 6B**. The adapted planktonic cells inoculated at 5 × 10^7^ CFU/ml (P) grew from 2 × 10^6^ to 6 × 10^6^ CFU/ml and converted L-malic acid during the first 10 days and then slowed down. The planktonic cells with oak chip (OP) also converted L-malic acid in 10 days, and then stagnated, due to their decrease in population after 10 days. The biofilm cultivated on oak (BO), inoculated at the equivalent of 5 × 10^7^ CFU/ml, performed complete MLF in 6 days. Interestingly, the biofilm released cells in wine, reaching 10^6^ CFU/ml on the 3rd day of MLF.

**Table 2:** Five conditions used to study *Oenococcus oeni*-oak-wine interaction.

Name	Inoculum	Oak
O	–	Untoasted oak chips (120 g/l)
P	Planktonic culture of adapted Sabo11 (5 × 10^7^ CFU/ml)	–
OP	Planktonic culture of adapted Sabo11 (5 × 10^7^ CFU/ml)	Untoasted oak chips (120 g/l)
BO	Biofilm 2-week growth of Sabo11 (5 × 10^7^ CFU/ml)	Untoasted oak chips (120 g/l)

*The wine is aligoté wine pH3.5 12% ethanol. The inoculum is absent, or a planktonic or biofilm culture of O. oeni strain Sabo11, the oak chip is immerged or not.*

**FIGURE 7:**
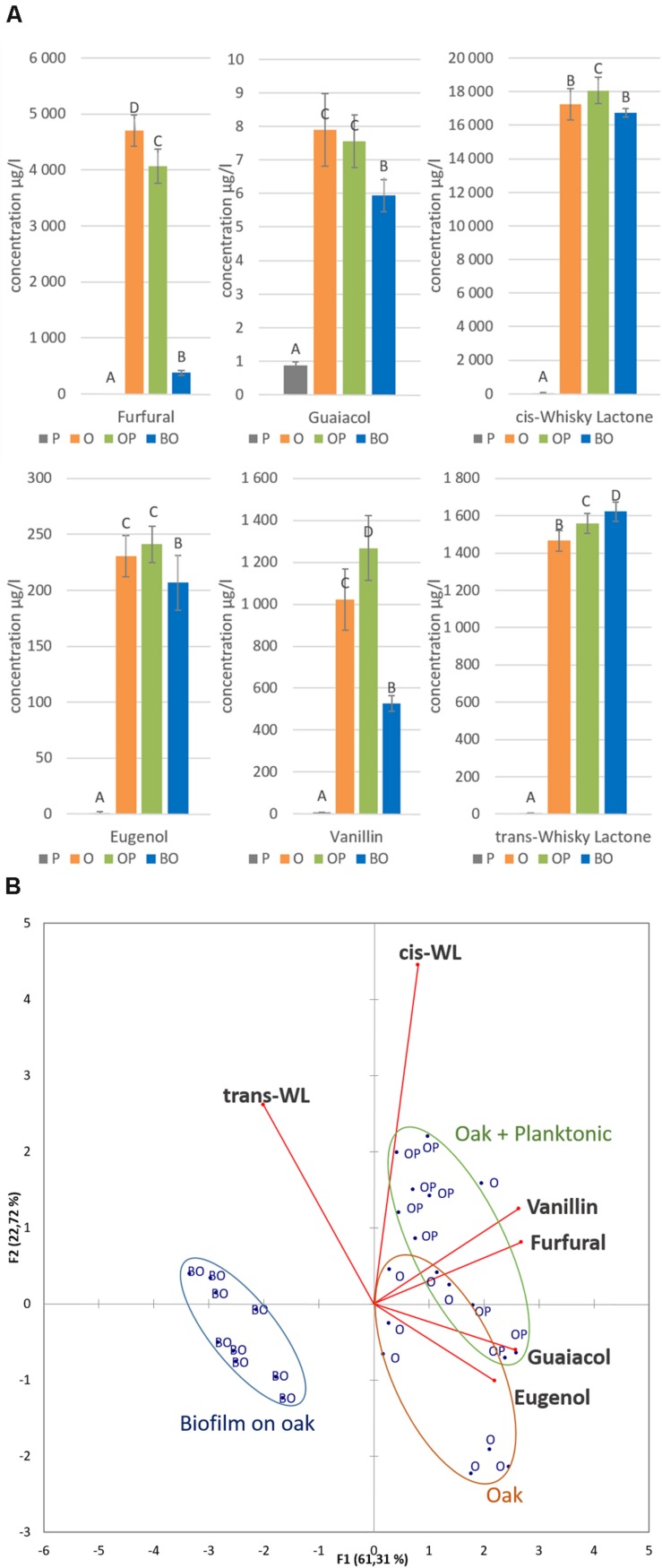
**(A)** HS-SPME-GC-MS analysis of six oak volatile compounds in wine after 1 month’s aging: furfural, guaiacol, *cis* and *trans*-whisky lactones, eugenol and vanillin. Four conditions were experimented, MLF by planktonic cells without oak (P in gray), oak chip immerged in wine without MLF (O in orange), MLF by planktonic cells with oak chip (OP in green), MLF performed by the biofilm on oak chip (BO in blue). Error bars represent the standard deviation of three biological and two technical replicates. **(B)** Projection of compositional data on principal components 1 and 2; the circled dots group the data of the six volatile compounds analyzed: oak alone (orange) oak with MLF (green) and biofilm on oak MLF (blue).

We continued to monitor MLF under these experimental conditions, and focused on the molecular interactions between *O. oeni*, wine and oak chips. To do this, the concentration of six oak volatile compounds in wine was assessed by HS-SPME-GC-MS analysis (**Figure 7A**). MLF performed by planktonic cells without oak (P) as a control showed that the six compounds did not come from the wine or the bacterial metabolism. Oak chips immersed in wine without cells to perform MLF (O) represented the reference compound transfer without bacterial metabolism. MLF with planktonic cells and oak chips (OP) influenced four compound concentrations, by increasing them (*cis*-whisky lactone, *trans*-whisky lactone, and vanillin) or decreasing them (furfural), whereas no significant difference was observed for guaiacol or eugenol. The biofilm under the oak chip condition (BO) released fewer oak volatile compounds than the O and OP conditions, except for the whisky lactones. The *cis*-whisky lactone levels of BO were similar to O, whereas the *trans*-whisky lactone level of BO was higher than the others. A principal component analysis was carried out to illustrate these aroma transfers from oak to wine as a function of direct inoculation process (**Figure 7B**). This representation shows that two components, F1 and F2, explain 84% of the variability of aroma concentrations. After only 1 month of micro-vinification, the biofilm lifestyle (BO) could be clearly distinguished from the planktonic lifestyle (OP). The presence of planktonic bacteria increased the vanillin concentration compared to the presence of the oak chip alone in the wine medium. This increase could be due to enzymatic activities, as described previously (de Revel et al., 2005; Bloem et al., 2006).

**FIGURE 8:**
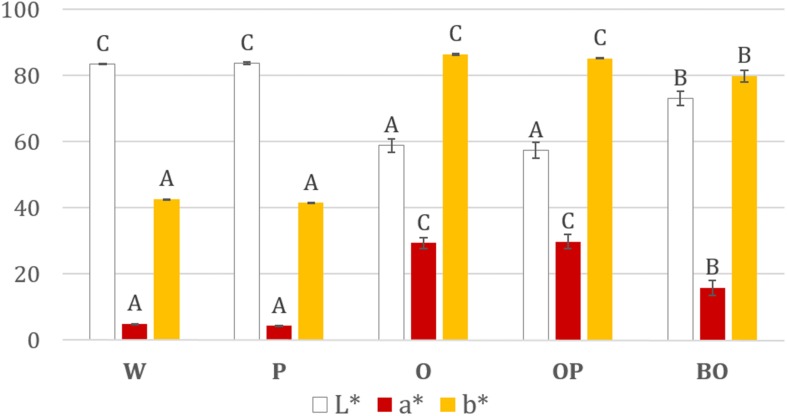
**L^∗^a^∗^b^∗^ parameters of the five wine conditions after aging for 1 month**. W, Wine; P, planktonic MLF; O, Oak; OP, Oak + planktonic MLF; BO, MLF by Biofilm on Oak **(Table 2)**. The error bars represent the standard deviation of three biological and three technical replicates.

Wine color, which is another enological parameter, was investigated in these micro-vinifications by measuring the chromatic L^∗^a^∗^b^∗^ values (**Figure 8**). Our study showed that MLF did not significantly change the color of wine (W vs. P). Likewise, there was no difference between oak wine with or without MLF (OP vs. O). Nonetheless, as expected, the impact of oak aging (O, OP) versus oak-less conditions (W, P) was an increase in the magenta (a^∗^) and the yellow (b^∗^) colors in the wine and a decrease of lightness (L^∗^). Finally, biofilms on oak chip (BO) reduced wine staining (a^∗^, b^∗^) and preserved lightness (L^∗^) compared to planktonic MLF wine with oak (OP).

## Discussion

In this study, culture based investigations and microscopy indicated that *O. oeni* actively colonized both steel and oak surfaces and formed agglomerates displaying the characteristics of biofilms. According to these findings, we investigated the biofilm development of *O. oeni* linked to its ability to perform MLF, a key step of winemaking. The study focused on: (i) the capacity of *O. oeni* to spatially organize in biofilm; (ii) the capacity of this biofilm to withstand the stress found in wine and to perform MLF; and (iii) the modulation of the organoleptic quality of wine by *O. oeni* biofilms developed on oak.

### Investigation of *O. oeni* Biofilm Development and Involvement in Resistance to Drastic Environmental Conditions

First, we highlighted *O. oeni* bacteria adhering to the wine material, which suggested the presence of potential biofilm. For the first time, *O. oeni* biofilm was developed on various materials including stainless steel and oak, which are used in winemaking with pumps, pipes, tanks, and barrels. Biofilm population is higher on oak than steel under the same growth conditions. This was expected, since stainless steel is frequently used in food processing to limit the adhesion of microorganisms (Hilbert et al., 2003), while wood has micro-topographical features and chemical structures that enhance bacteria adhesion (Mariani et al., 2007).

The biovolume of *O. oeni* biofilm assessed with CLSM was 4 × 10^5^ μm^3^ from 20-h growth and stayed the same until 40 h. This biovolume was close to those obtained from other LAB such as *Lactobacillus casei, Lb. plantarum*, which are around 2 × 10^5^ μm^3^ at 48 h (Rieu et al., 2014), although *O. oeni* has a slower growth rate (μ_max_ = 0.11 to 0.17 h^–1^) compared to these LAB, e.g., 0.6–0.11 h^–1^ for *L. casei* and *Lb. plantarum*. Therefore, under these confocal microscopy conditions, *O. oeni* biofilm growth reached a level similar to that of other LAB species known to form biofilms.

Biofilm lifestyle is well known to protect bacteria from harsh environmental conditions. In our model, cells from *O. oeni* biofilms were much more resistant than planktonic ones, in agreement with findings on the biofilm cells of *Lb. plantarum* that exhibit improved resistance to ethanol (Kubota et al., 2008, 2009).

In order to understand how biofilm allows cells to withstand environmental stresses, the expression of genes encoding proteins involved in the stress response of *O. oeni*, i.e., Lo18, GroEL and ClpL1 and CFA synthase was investigated (Guzzo et al., 1997; Beltramo et al., 2004, 2006; Grandvalet et al., 2008; Maitre et al., 2014). These studies revealed that stress-related genes are often overexpressed in biofilm *E. coli* populations compared with planktonic cultures, even in the absence of environmental stress (Schembri et al., 2003; Domka et al., 2007). Under our culture conditions, the stress-gene expression observed was lower in biofilm than in planktonic cells. This could be due to the kinetics of these genes’ expression as a function of the growth stage in the biofilm. Indeed, stress proteins might have been produced already and fulfilled their protective role. Consequently, the biofilm could preserve its resources and energy (Beloin and Ghigo, 2005). Another explanation is related to the fact that gene expression analysis is generally global, considering the biofilm as a whole. But biofilms are described as heterogeneous populations with local spatiotemporal patterns of gene expression. This overall measure gives us an average picture of the actual gene expressions, which likely smooths out differences between cells (Coenye, 2010; Mielich-Süss and Lopez, 2015). Cells in different metabolic states within the biofilm characterize this heterogeneity. Indeed, a study on *Bacillus subtilis* biofilm cells showed that cells multiply on the surface layer, whereas in the middle of the biofilm cells produce an extracellular matrix to reinforce the biofilm structure (Vlamakis et al., 2008; Mielich-Süss and Lopez, 2015). Despite the low stress gene expression observed, the *O. oeni* cells in biofilm exhibited increasing resistance to stress, suggesting that one or more other mechanisms contribute to this tolerance. We can conclude that this observation favors the involvement of the biofilm EPS matrix, even if *O. oeni* cells in biofilm remain reactive to stress by inducing stress gene expression.

### *Oenococcus oeni* Biofilm is Able to Perform MLF and Modulate the Organoleptic Properties of Wine: an Alternative to Adapt MLF Starters

Our study shows that *O. oeni* cultivated in biofilm kept its malic acid conversion ability under drastic conditions without any prior adaptation, due to the greater survival of biofilm cells and the diffusion of malic acid through the EPS matrix. A previous study using adapted planktonic cells (ATCC –BAA 1163) demonstrated the consumption of malic acid in 16 days (Beltramo et al., 2006). However, comparing different studies is extremely difficult since their conditions also differ. Indeed, a slight change of ethanol concentration (0.5%), pH (0.1 unit), or temperature (5°C) can change the outcome of the study. *O. oeni* biofilm cell resistance and activity seem to be close to those of immobilized cells, which are the subject of intense research. Indeed, several experiments have performed MLF with *O. oeni* immobilized on various surfaces: fibrous cellulose sponge, corn cobs, grape skins and grape stems (Genisheva Z. A. et al., 2014), resulting in varying degrees of success. The common trait between these studies is increased *O. oeni* cell resistance when immobilized, compared to the planktonic reference (Genisheva Z. A. et al., 2014). However, immobilized cells cannot be considered as a proper biofilm since cell growth, cell–cell interaction, and multifunction matrix are highly specific to the biofilm phenotype (Davey and O’toole, 2000; Hojo et al., 2009; Flemming and Wingender, 2010; Coenye, 2010).

Subsequently, our study focused on the modulation of oak flavor compounds in the wine by biofilm grown on oak. *O. oeni* glycosidase activity has been shown to release aromas from oak (Bloem et al., 2008), including vanillin (Bloem et al., 2006). Although oak aroma compounds are sought for increasing wine sensory properties, it is interesting to be able to modulate their concentration in wine (Duval et al., 2013). In our study, white wine whose MLF was carried out by biofilm on oak also exhibited these same differentiations in the aromatic profile, marked by a decrease of oak aromatic compounds (*cis*-whisky lactone, vanillin, eugenol, guaiacol, furfural). Interestingly, in the same wine, *trans*-whisky lactone was present at higher concentrations, suggesting that wood/wine interactions under the action of *O. oeni* biofilm could modulate the aromatic complexity of wine. This could be explained by the matrix covering the oak surface and acting like a filter (Dunne, 2002). These compounds may be bound with the EPS or even be converted by biofilm enzymes. Since the sensory contribution of *trans*-whisky lactone is slight (perception threshold of 110 μg/l), such aroma analyses clearly highlight the potential interest of *O. oeni* biofilms for monitoring the oak aging of wines to obtain the fine-tuned extraction of wood aromas. In the same way, wine color obtained during aging is modulated by the presence of biofilm on oak. As the biofilm modulates the organoleptic profile of wine, we suggest a retention effect of the matrix with possible interaction between EPS and wine molecules, such as macromolecules classified as anthocyanins and tannins (polyphenols).

## Conclusion

*Oenococcus oeni* biofilm could be considered as a novel approach for performing MLF, and as an alternative way of adapting MLF starters to wine stress. Moreover, biofilm can modulate the organoleptic profile of the wine. These results were obtained only with unheated wood, and more in-depth investigations are needed to account for the general use of oak aging by winemakers.

## Author Contributions

AB performed all the experiment. RB and AC contributed to obtain the confocal microscopy data. CC and RG supervised the experiments related to organoleptic profiles. HA took part to wine production. SW and JG conceived the work and supervised the experiments. All the authors contributed to writing the paper.

## Conflict of Interest Statement

The authors declare that the research was conducted in the absence of any commercial or financial relationships that could be construed as a potential conflict of interest.

## Footnotes

1 www.jouy.inra.fr/mima2
